# New products

**DOI:** 10.1155/S1463924689000386

**Published:** 1989


Journal of Automatic Chemistry Vol. 11, No. 4 (July–August 1989), pp. 179–190

### New products

### Amino-acid analyser

The System 6300 Amino-Acid Analyser from Beckman Instruments is now available with the System Gold Data System. This system allows nutritionists and biochemists to simultaneously collect data from instruments while reanalysing previously collected data. Applications include the diagnosis of inborn errors of metabolism, monitoring of dietary and follow-up treatment, the nutritional assessment of pre-surgery and trauma patients, and follow-up treatment of these patients.

The 6300 offers performance equal to dedicated systems. Amino-acids are derivatized with ninhydrin in a microvolume tubular reactor set at an ideal 135°C. This patented 100 μl reactor is packed with diamond dust to optimize the reaction, and the small volume ensures greater quantitative accuracy than can be achieved with larger capacity reactors in which diffusion and consequent loss of resolution may occur.

An in-line conductivity sensor ensures complete filling of the metering loop through which sample flows, and allows reliable and precise sample delivery to the column.

The stainless-steel analytical columns are prepacked with spherical ion-exchange resin—a lithium buffer form for physiological analyses, and a sodium buffer form for hydrolyzate analyses. High-pressure operation up to 3000 p.s.i. permits fast, accurate analyses.

The manufacturer offers buffers, reagents and standards specially formulated for the system. In addition, Beckman provides extensive after-sales support with user training programmes and applications assistance from the company's research scientists and technical library.

*For more information contact Beckman Instruments, Inc., 2500 Harbor Blvd., Fullerton, California 92634-3100, USA. Fax: 714 870 8083.*

### Robotic sampler senses liquid level

Several advanced features have been added to Beckman's Biomek robotic laboratory workstation for the automation of microtitre plate work.

An interchangeable pipetting tool – the P200L – is a syringe device which pipettes liquids with a volume range 10–20 microlitres with 1 microlitre increments. The P200L's liquid level sensing system, which uses disposable plastic tips to eliminate carry-over, finds the meniscus of sample liquids, avoiding tip over-immersion and therefore eliminating contamination. The sensing system functions with aqueous and non-aqueous liquids and verifies tip pick-up by the tool. The P200L can cope with a range of depths, from those of microtitre plate wells to test-tubes.

The Biomek 1000 PC-based control software has also been upgraded for use with the new Biomek Sideloader (SL) which automatically loads labware onto the workstation. The SL provides several hours of totally unattended operation for many applications, including dilution work and routine assays such as ELISA and receptor binding. An additional software enhancement means that the normal operation of Biomek can be interrupted to run an external program to organize files, transfer data, perform calculations and switch external devices.

*Further information from Beckman, Progress Road, Sands Industrial Estate, High Wycombe, Buckinghamshire, UK. Tel.: 0494 441181.*

### Gas analysis

The Bioquad gas analysis system from Spectramass, which is designed for the monitoring of fermentation and other biological processes, has been attracting a good deal of interest since its introduction last year.

New options include a more flexible, spread-sheet-compatible software package for data logging and data manipulation, making the gas analysis data more readily available for combination with other process measurements.

Improvements to membrane probe technology now permit the easy analysis of dissolved gases and volatile components present within a fermentation broth, and the new design now permits the multiplexing of probes so that any combination of membrane probes, inlet and exhaust gas streams can be handled by one Bioquad control unit.

*For more information contact Spectramass Ltd, Radnor Park Industrial Estate, Congleton, Cheshire CW12 4XR, UK. Tel.: 0260 279531.*

### Particle size analysis

Labcon are now offering a quick solution to particle size analysis. Their Epson Pine system, which is mounted in a splash proof case, is pre-programmed to control both the sieve shaker and electronic balance and to produce a rapid print out of analysed data. Key features of the package include simple screen-prompted operation; onscreen graphic display; memorized sieve sizes and weights; results storage; remote or inbuilt printer options; compatibility with all sieve shakers and many commonly used balances with a bidirectional RS232 interface; additional software options to customers' own specifications.

*Further details from Labcon, 24 Northfield Way, Aycliffe Industrial Estate, Newton Aycliffe, County Durham DL5 63J, UK. Tel.: 0325 313379; fax: 0325 311044.*

### Gas-detection instrumentation in response to COSHH

In response to new COSHH legislation, Bruel & Kjaer have announced a multi-gas monitor which provides quantitative analysis of up to five components and water vapour in an air sample.

0309–1902/89 $3.00 © 1989 Taylor & Francis Ltd.

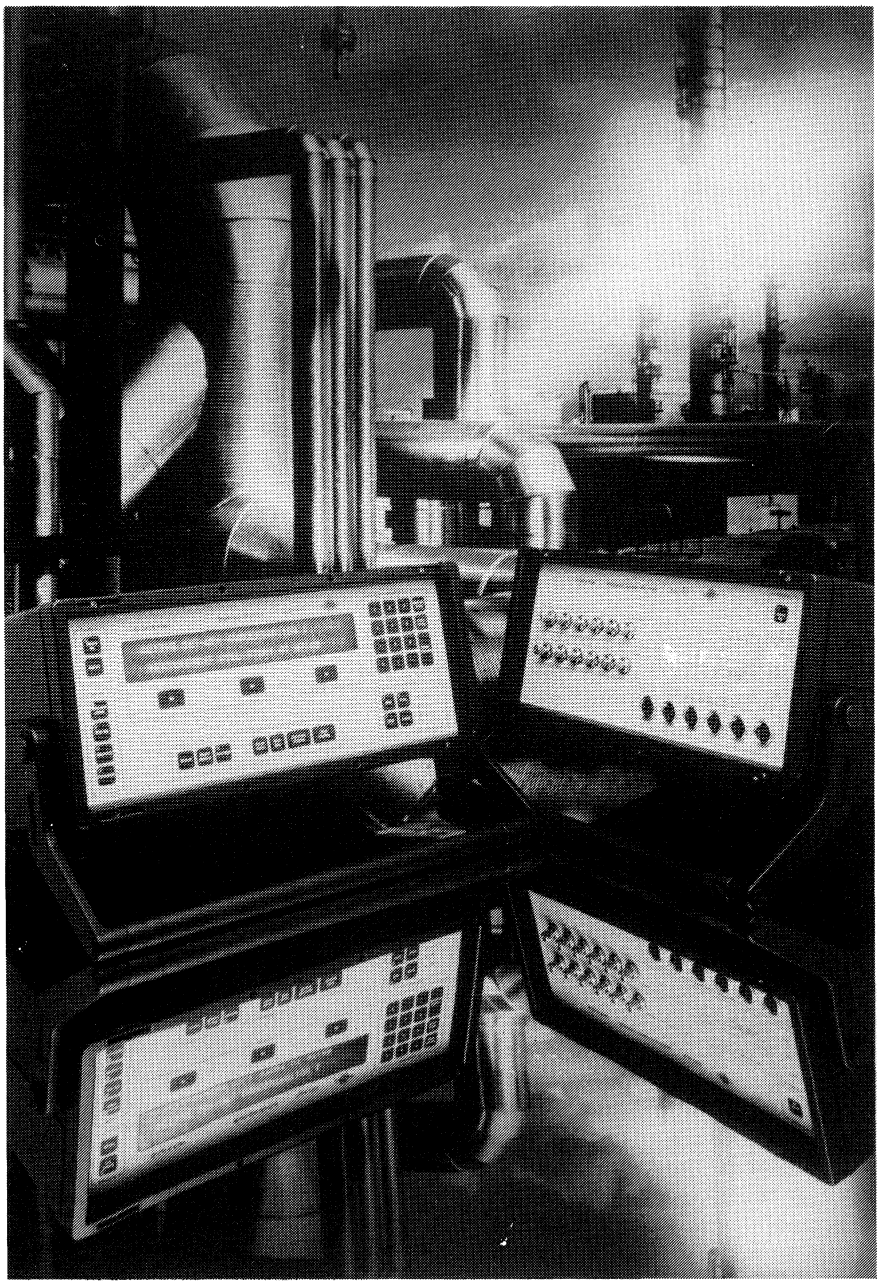

*Bruel & Kjaer's gas-monitoring equipment, which has been designed to help employers in the UK to comply with recent government safety legislation. The instrumentation can measure virtually any gas which absorbs infra-red light.*

In October 1989 UK employers become subject to legislation, based on EEC directives, related to the control of hazardous substances. The legislation is aimed at improving air quality, and creates a need for precise measurement of gases and vapours by both employers and the government.

The Type 1302 Multi-gas Monitor, which is the first product on the market from the company's Manchester-based Gas Analysis Division, is a portable, easy-to-use instrument designed to perform the measurement tasks required by the legislation. The measurement principle is based on photoacoustic infrared detection, a technique which offers advantages over existing methods in terms of speed of response, sensitivity, calibration interval, and immunity to interferents.

The Type 1302 can be used to measure virtually any gas which absorbs infra-red light, and can be equipped with the appropriate optical filters to enable measurement of the concentrations in an air sample of up to five component gases and water vapour. The detection threshold is gas dependent and of the order of $10^{-3}$ parts per million. Addition of the Type 1303 Multipoint Sampler and Doser enables air sampling at six locations within 50 metres of the instrument, with delivery of tracer gases if required.

A special feature of the analyser is compensation for water vapour, and any other known interferent, by the addition of optical filters. Once the filters are installed, the instrument is zero-point and span calibrated and holds its calibration for around three months – this is significantly longer than chromatographic and spectroscopic alternatives. Re-calibration is a simple procedure directed by on-screen prompts; and comprehensive self-testing aids performance reliability.

The 1302 can be set up and used by personnel with no technical expertise and any sequence of measurement functions can be programmed to be automatic. Results are stored in memory, and a number of stastistical functions are built in. RS232C and IEEE488 interfaces are provided for remote control and output of results to any standard text printer.

Applications will include engineering, chemical, pharmaceutical, mining, oil and gas, water, building, transport and waste disposal industries, medical environments, and fire-fighting authorities.

*For further information contact Bruel & Kjaer (UK) Ltd, 86 East Road, Longsight, Manchester M12 5GY, UK. Tel.: 061 224 7590; fax: 061 248 6140.*

### Gas analysis brochure

Spectramass have recently published a short brochure giving details of their complete range of products for vacuum elucidation, leak detection and gas analysis. The colour leaflet covers RGA, environmental monitoring, process control, QC and research applications.

*Copies from Spectramass Ltd, Rednor Park Industrial Estate, Congleton, Cheshire CW12 4XR, UK. Tel.: 0260 279531.*

### GC-TALK

GC-TALK links the Model 8600 and 8700 has chromatographs to an Epson or IBM (or compatible PC) for transfer of methods, reports, chromatographic data and automation control parameters. Data can be stored in the PC for later reprocessing and review. It is based on GEM software, with mouse control and pull-down menus for ease of use. Automation control allows completely unattended operation of the gas chromatograph for fully automated analyses and data storage. In addition, instrument can be remotely started by the PC during automation queues. Hard copy can be produced on the PC's printer, or the GC printer/plotter; and chromatographic data and reports may be exported to commercial software, such as word processors or spreadsheets.

*For further information contact Perkin-Elmer Ltd, Maxwell Road, Beaconsfield, Buckinghamshire HP9 1QA, UK. Tel.: 0494 676161; fax: 0494 678324.*

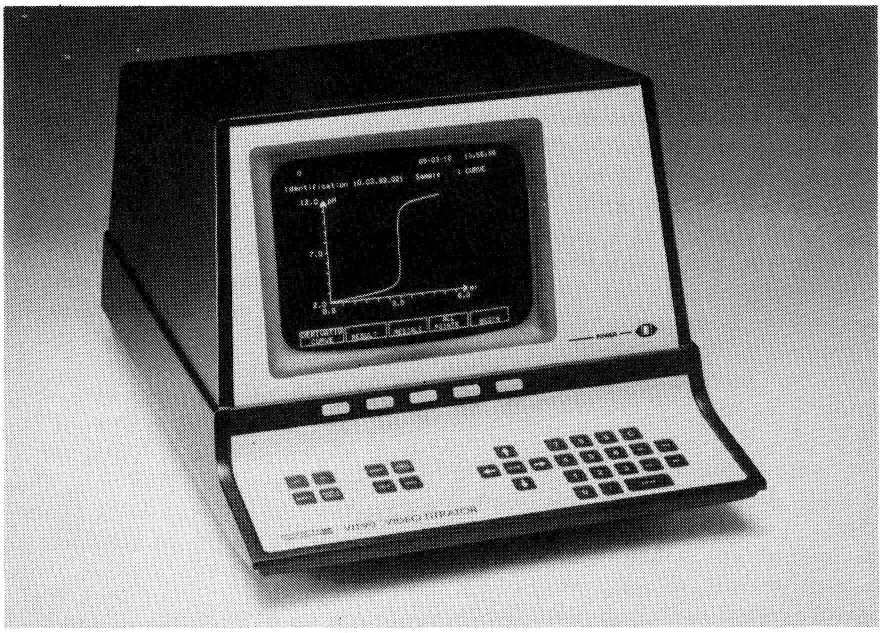

*Users of the TitraLab High Performance Titration Laboratory requested a number of enhancements of the system. Radiometer Analytical A/S have recently improved the software in the VIT90 Video Titrator – the 'brain' of the system.*

### Quality control

Software for Beckman's Real Time Quality Control (RTQC) system allows users instant access to months of quality control data. The package deals with laboratory QC data from all instruments within the clinical facility and can monitor quality on any manufacturer's instrumentation or control materials.

RealTime software is an intralaboratory IBM MS-DOS based quality assurance system which eliminates hours of paperwork and calculations, and maintains permanent records of out-of-control situations with historical corrective actions.

*Further details from Beckman, Progress Road, Sands Industrial Estate, High Wycombe, Buckinghamshire, UK. Tel.: 0494 441181.*

### Titration

Radiometer Analytical A/S have announced a series of enhancements to the software in the VIT90 Video Titrator. With an increasing number of samples, identification is crucial. VIT90's new sample stack includes full information on up to 60 individual samples: numeric and/or alphanumeric ID, weight or volume, plus a sample-specific factor, for example to correct for drying loss. An easy-to-use 'spelling' display enables the operator to enter alphanumeric sample data rapidly.

Analysis of water often includes pH, conductivity, titration, and measurement with Ion-Selective Electrodes. With TitraLab, combinations of these can be performed automatically. The new VIT90 has direct serial communication with the autoranging CDM83 Conductivity Meter; consequently, it works within seven decades without operator interaction.

Calculation facilities have also been expanded. In addition to a built-in formula library with all standard formulas, the user can specify and store eight further formulas for each method. Normal operators as well as 'Log' and 'Exp' functions, can be used.

Global variables can be used for transferring results from one titration to another. With this facility, the result of a titre determination can be incorporated in all methods using this titrant concentration for calculation of results. Statistics with mean value and standard deviation are available and can be reset automatically. Thus, requested means of duplicates or triplicates are easily obtained.

Also titration curves can now be reprocessed electronically. This facility is especially useful during general methods development. Results can be reviewed and alterations made: so all curve inflections are detected. Even the weakest equivalence points are never lost.

Reaction kinetics measurement often includes calculation of reaction rate. With the new TitraLab, a user-selectable calculation window can be used to determine maximum and means reaction rates for the desired parts of the curve. Correlation coefficent, statistics, and user-programmable formulas are available.

*For more information contact Radiometer Analytical A/S, Krogshojvej 49, DK 2880 Bagsvaerd, Denmark. Tel.: 45 31 696311; fax: 45 44 490011.*

### Electric shock treatment opens cells to genetic manipulation

Probably the most significant recent advance in biotechnology instrumentation has been the development to the twin techniques of electro cell fusion and electroporation. These provide an alternative to tissue culture for creating hybrid cells or inserting genetic material. A range of top-quality instruments for these techniques is now available in the UK from Biotech Instruments of Luton.

By exposing animal, plant or bacterial cells to carefully applied, high voltage, pulsed electric fields, pores are opened up in cell membranes through which cells can be fused together (electro cell fusion) or genetic material inserted (electroporation). This provides a simple, clean, and easily controlled means of performing cell manipulations, and is especially useful when dealing with cells which resist the insertion of genetic material in tissue culture (for example wheat, corn and other grain cells).

The equipment developed and manufactured by Biotechnologies and Experimental Research Inc (BTX) of San Diego, California is widely recognized as the most advanced and effective for this type of work. The BTX range is now available in the UK exclusively from Biotech Instruments, who can provide detailed advice, based on an extensive data-base of published work, on the optimum way of handling many different cell types.

The Electro Cell Manipulator 200, a sophisticated system for cell fusion and electroporation of human, animal and plant cells, features a 1 MHz radiofrequency generator for rapid and consistent cell alignment. Its low impedance design provides maximum voltage transmission, and precise, high voltage, square wave pulses are selectable in 1 μsec pulse width increments, with automatic multiple pulsing capability. Hybridoma yields of mouse and human cells have been shown to be 10 times those obtained by conventional methods, and the rapid fusion of plant protoplasts is also performed at much higher efficiencies.

Electroporation of micro-organisms requires very high electrical field strengths, and for this the Transfector 100 is ideal. High voltage electric pulses enhance the transformation frequencies of intact microbial cells, providing a highly efficient means of transforming most popular strains of *E. coli, Streptococcus lactis* and many other micro-organisms, including Gram-positives.

A wider range of capacitance settings, ranging from 50 to 3150 μF in 50 μF increments, is available with the Transfector 300. This gives the researcher the ability to fine-tune pulse lengths to optimize transformation efficiencies.

The main feature of these systems is a graphic pulse analyser, the Optimizor, which monitors chamber conditions during electro cell fusion and electroporation to a very high level of accuracy. A liquid crystal display shows a graphic trace of the pulse going into the chamber, together with measurements and parameters. In this way the exact chamber field strengths are indicated instantaneously, so that optimal experimental conditions may be established.

Safety has been a prime consideration in the design of BTX systems. Safety features include full protection against operator shock and short circuit, which is an essential safeguard as very high voltages are used. An isolator guards against accidental exposure to the electrodes during operation.

A choice of nine different chambers, both disposable and re-usable, are offered, in 14 sizes ranging from a 20 μl microslide to a 75 ml production chamber.

*More information from Biotech Instruments Ltd, 183 Camford Way, Luton, Bedfordshire LU3 3AN, UK. Tel.: 0582 502388; fax: 0582 597091.*

### High-performance, high-resolution capillary GC

Since its launch in 1983, the HP 5890A has proved a highly successful gas chromatograph. Hewlett-Packard have launched a new series II version, which represents the state of the art in analytical GC technology.

Chromatographic performance is increased by the facility for on-column capillary injection. The on-column capillary inlet option is compatible with the HP 7673A automatic injector and sampler and allows automated methodologies using on-column capillary injection to be developed, without compromising on flexibility or ease of use.

Electronic pressure control of the on-column inlet means that column head pressure, as well as inlet temperature, may be defined from the keyboard. This gives increased retention time reproducibility and the ability to separate complex mixtures.

Pressure programming of the column head provides more accurate results, as compounds are analysed at lower temperatures and therefore with less thermal degradation. Analyses are faster, and cryogenic cooling reduces the time between sample runs.

The oven temperature range on the Series II has been extended to 450°C. With the on-column inlet, this allows high boiling-point compounds to be analysed with ease. Such applications as the analysis of C-120 in waxes, and of steroids and triglycerides in biosamples, may be performed automatically, as may petroleum applications where higher temperatures are required for accurate characterization of heavy fractions.

The capacity of the HP 5890 Series II is doubled by the use of two valve-driver channels, which reduce the cost of valve configurations. Additional heated zones provide increased flexibility.

Automatic control is further enhanced by an events monitor within the GC which allows time-programming of thermal conductivity detector sensitivity, detector signal switching and multiple valve events. Storage of GC setpoint makes the unit easier to use as a stand-alone device.

Current users of the HP 5890A may upgrade their systems to full Series II

specifications, to incorporate the new features and options while retaining existing methods and processes.

*Details from Analytical Products Group, Hewlett-Packard Ltd, King Street Lane, Winnersh, Wokingham, Berkshire RG11 5AR, UK. Tel.: 0734 784774.*

### Wallac sale to Bio-Orbit

The luminescence product line marketed by Wallac OY (a member of the Pharmacia group) has been acquired by Bio-Orbit OY, a company specifically formed for this purpose. A wholly owned subsidiary, Bio-Orbit (UK) Ltd, has also been established.

The new companies intend to make rapid bacterial cell counting as widespread in routine quality control in production areas as it is in quality-assurance laboratories, which is timely in view of the current public interest in bacterial infection. A rapid assay of microbiological conditions is an almost essential requirement for anyone with a responsibility for standards of any ingestible product or service, where the mere hint of contamination leads to the media demanding instant confirmation or denial. With Bio-Orbit's luminescence method of counting bacteria, the results can be verified within minutes (as opposed to hours or days required with incubation on culture plates) which means that process or product contamination may be determined at an early stage of production, avoiding subsequent wastage and possible damaging speculation of a bacterial outbreak incident.

*Details from Bio-Orbit OY, Mustionkatu 2, SF 20750 Turku, Finland. Tel.: 358 21 363 666; fax: 358 21 363 150.*

### Centrilab

A new company has been formed in Ringwood, UK, to manufacture and distribute bench-top centrifuges. 'Centrilab' is run by Simon Ellis and David Prevett, who have taken over the production and sales of low-cost, compact centrifuges from Ellisend Centrifuge Services.

The company was formed to fill a gap in the market for simple, high-performance units across many industries. Small centrifuges are required by pathology laboratories, general practitioners, veterinary practices, food processing businesses, petro-chemical and chemical works, marine and electronic industries. Centrilab offer a range of UK-manufactured centrifuges which are ideal for industrial use as a simple laboratory tool.

An agreement has been reached with JS Pathology PLC who offer a leasing service on Centrilab centrifuges as part of their drug-testing service.

Covering such a wide range of industries, Centrilab's first design has been developed with customizing in mind so that, for example, special heads can be readily supplied to suit many sizes and types of sample tube.

*More information from Centrilab at Kingsbury House, Friday Cross Mews, Christchurch Road, Ringwood, Hampshire BH24 1DG, UK. Tel.: 0425 480455.*

### Process monitoring of industrial effluent

By monitoring sewage and waste effluents for chemical oxygen demand, COD, potentially dangerous levels of contaminants can be detected and diverted in time to maintain effluent concentrations within safe limits. A new process COD analyser by Ionics Ltd, designated the Model 304, is intended for industrial and municipal sewage and waste real-time monitoring to ensure compliance with water authority requirements. Whereas many laboratory methods on grab samples can take hours and miss possible spillages, the COD analyser is designed to operate continuously and provide reliable on-line monitoring.

The Model 304 COD Analyser employs the acid dichromate open reflux method. Chosen for its high efficiency in oxidizing a variety of organics in water, the acid dichromate open reflux method involves refluxing at 150°C for a fixed period of time (normally 2 hours, but this is user programmable). The COD measurement is then measured colorimetrically, providing a rapid and sensitive determination of dichromate concentration. The amount of dichromate consumed is directly proportional to the sample COD Automatic calibration and zero-check are standard.

*For further information contact Ionics UK Ltd, Carrington Business Park, Carrington, Urmston, Manchester M31 4DD, UK. Tel.: 061 776 4550; fax: 061 777 9630.*

### Yokogawa introduces integrated factory-wide control system for small processing operations

A new cost-effective distributed control system, designed to offer smaller industry manufacturers the same processing benefits previously available only to larger plants, is now available from Yokogawa Electric, international supplier of control systems.

The MXL (Micro Excel) Distributed Control System is a completely integrated, factory-wide control system based on micro-level computing, but providing the same processing benefits normally associated with systems served by midrange or mainframe computers.

The MXL is designed for the real-time production process control of a plant's batch, discrete and continuous processing operations. The basic system consists of an operator station, a field control unit and a communication bus that can manage everything from raw materials, advanced batch-sequence and production-process control to shipping.

As orders come in to a plant, the MXL determines how many products or activities must be initiated. It tracks inventory to ensure that enough resources are available and indicates when supplies are low. The MXL is capable of memorizing a number of pre-set recipes or production processes and will aid in determining when maintenance checks or clean-up procedures should start. Finally, when the product is completed, the MXL will track packing (where each package should be shipped, and which shipment will contain which packages).

Ready-to-use application packages and 'fill-in-the-forms' engineering, as

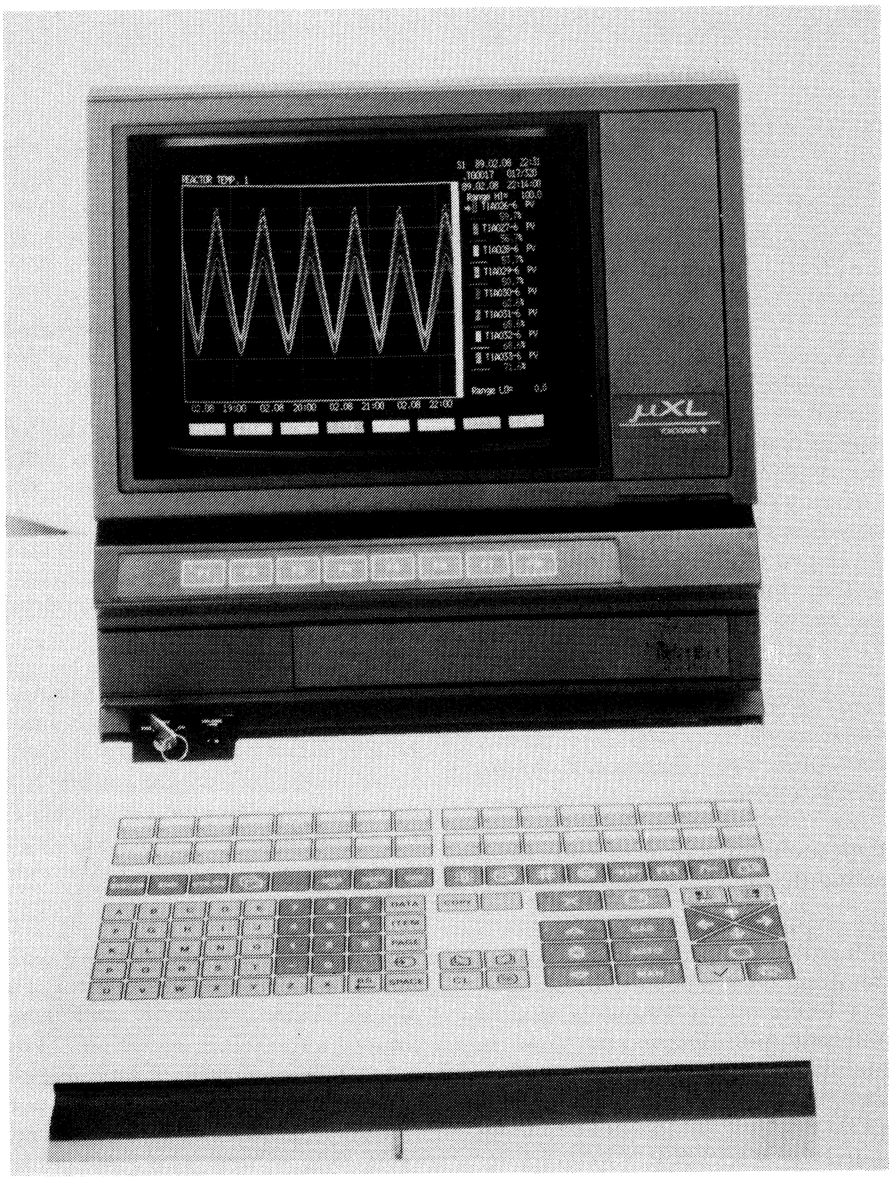

*The MXL Distributed Control System from Yokogawa Electric Corporation. Ready for shipping, the system costs from $40 000.*

well as BASIC and graphic builder software, mean low engineering costs, flexibility and use-configurability. Expandability is another important feature of the system – its fibre optic data link can span the largest factories.

The basic MXL system consists of two PC-like control units, one central and one for field use and a communications arm. Expansion is achieved by adding more control units.

Based on the same technology that Yokogawa developed for its Centum system, the MXL incorporates various feedback and sequence control functions, including simple PID control, dead time compensation, feedforward control and scheduled start-up.

In addition, the system contains a real-time BASIC programming environment which can handle such sophisticated management tasks as real-time management reporting, logging, system interfacing, recipe management and data analysis.

*For further information contact Yokogawa Electrofact B.V. Headquarters Radiumweg 30, 3812 RA Amersfoort, The Netherlands. Tel.: 31 33 641611; fax: 31 33 631202.*

### Chromatography software on VAX

Beckman's PeakPro chromatography software is now available to run on VAX computers. Proven in use since 1986, when it was first introduced on Hewlett-Packard computers, PeakPro greatly simplifies the work of the chromatographer. Easy-to-understand full colour menus and 'user friendly' conversational prompts, guide the operator through a range of functions.

Data reduction and reporting routines include internal and external standards and normalized per cent with multi-level calibration. Real-time plotting is possible while data are being acquired. The package also contains many options for expanding and comparing stored chromatograms; and results summaries for multiple analyses may be produced for comparing and identifying trends.

PeakPro also offers full compliance with GLP/GMP guidelines. All data are protected from being changed or purged except by authorized users, and any such activity creates an automatic entry in the system log. Furthermore, full documentation of all analyses is provided. Test results, summary reports, system suitability and audit trail reports are easily called on screen or printed on command.

Although the basic PeakPro package is an economical solution for smaller laboratories, the modular design of its hardware and software means that the system can grow with the needs of the laboratory – the basic system can readily be expanded into a full-scale CALS Lab Manager LIMS system.

*For further information contact Beckman Instruments (UK) Ltd, Progress Road, Sands Industrial Estate, High Wycombe, Buckinghamshire HP12 4JL, UK. Tel.: 0494 441181; fax: 0494 461935.*

### Chemical analyses documented for metal plating industries

A series of 15 chemical analyses — especially documented for use in metal plating and coating process laboratories – is now available, free of charge, from the Radiometer UK.

Close monitoring of the concentration of metals and impurity levels in plating solutions is essential to achieve and maintain optimum process efficiency, cost-effectiveness and end-product quality. All 15 analyses employ potentiometric titration, a field in which Radiometer has long offered a wide range of instruments and ancillary equipment.

The procedures determine cadmium, chromium, copper, gold, lead, nickel, silver, tin and zinc in plating solutions and also sulphuric acid and aluminium together in anodizing solutions. Other analyses within the 15 are aimed at controlling boric acid, chloride and cyanide levels in the plating baths.

*The metal plating analyses are available as the 'Plating Solutions Package' from The Analytical Division, Radiometer Ltd, The Manor, Manor Royal, Crawley, West Sussex RH10 2PY, UK. Tel.: 0293 517599.*

### Autosampler for ICAP spectrometers

Thermo Jarrell Ash's TJA-300 autosampler, which has 300 sample tube positions, allows large sample workloads to be run without constant attention. The TJA-300 incorporates decision-making logic for true unattended operation. The TJA-300 accommodates automatic profiling, automatic standardization, limit checking of samples, and quality-control checking with the option of reprofiling and/or restandardizing upon failure. All rinse and sampling times are computer controlled through menus selections in the ThermoSPEC software.

When coupled to the TJA Prep Station and sample mixing vessel, the TJA-300 can be used to automatically dilute and mix samples according to a user-generated method. After dilution, the samples are automatically delivered to the plasma.

The TJA-300, with its optional overflow sampling station and external warning system, can be used to automatically sample a stream piped to the autosampler with its sipper probe. The sampled stream passes to a flow-through mixing vessel for dilution, followed by delivery to the sample introduction system of the spectrometer. The quality-control routines of ThermoSPEC software can be used to monitor control levels in the stream. Any out-of-control results can trigger automatic recalibration of the spectrometer and reanalysis of the controls.

*More information from Thermo Jarrell Ash Corporation, 8E Forge Parkway, Franklin, Massachusetts 02038, USA. Tel.: 508 520 1880.*

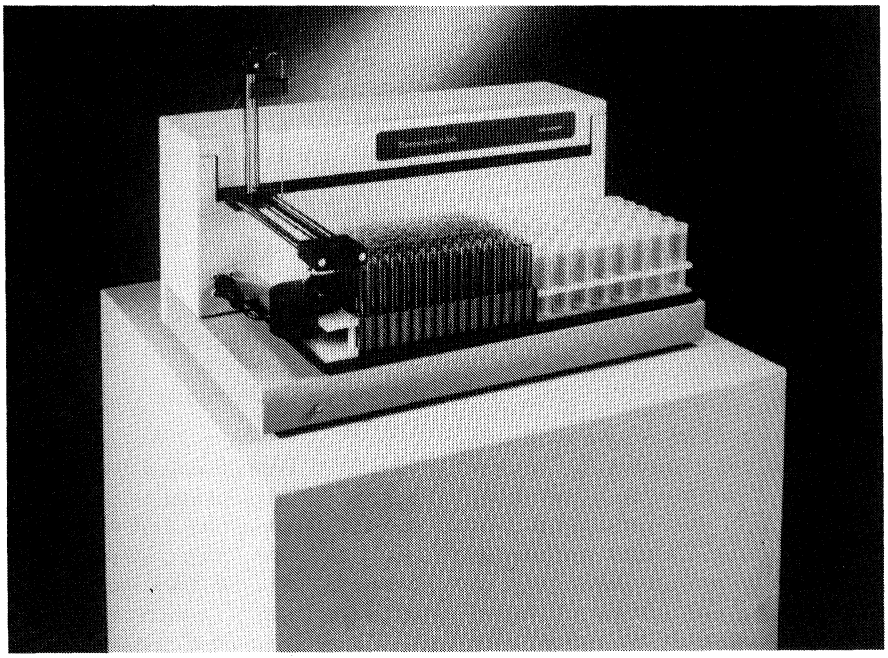

*The TJA-300 autosampler for ICAP spectrometers.*

### Factory management

'FIP' is a new digital communications system for factory management. Factory Instrumentation Protocol is an industrial network, or bus, intended to replace point-to-point wiring, connecting sensors, actuators and regulators. It is also an open system, so it provides a means of connecting the components of the modern automated factory – processing units, programmable controllers and robots.

In operation, FIP improves the general behaviour of control systems, ensuring that all components receive coherent information. Once installed, the system needs little maintenance, and it is easily upgraded to adapt to changing conditions.

The system acts as a real-time distributed data-base, providing data to management and maintenance services. FIP uses a 'broadcasting' topology, in which each device connected is a potential consumer of information broadcast on the bus. It can be up to 2000 m in length and can support as many as 256 stations. FIP was developed by a non profit-making consortiuum of French companies, and has been tested in sites which include hydro-electric power stations, oil refineries and manufacturing plants.

*Details from Jean-Pierre Bardinet, Club FIP, Ensem, BP 850, F 54011 Nancy Cédex, France. Tel.: 83 32 3901.*

### Multi-channel pipettes

A range of multi-channel pipettes that take the 'hit-and-miss' out of volume setting was recently launched by Flow Laboratories. The pipettes feature a preset volume control and digital display, which make incorrect setting virtually impossible. The pipettes have a colour-coded thumb button which is used to dial-in the required dispense volume – the volume is then displayed in an integral window.

The pipettes are available with four-, eight- and 12-channels. All models in the range are lightweight, reducing operator fatigue and feature individually replaceable tip cones, keep-

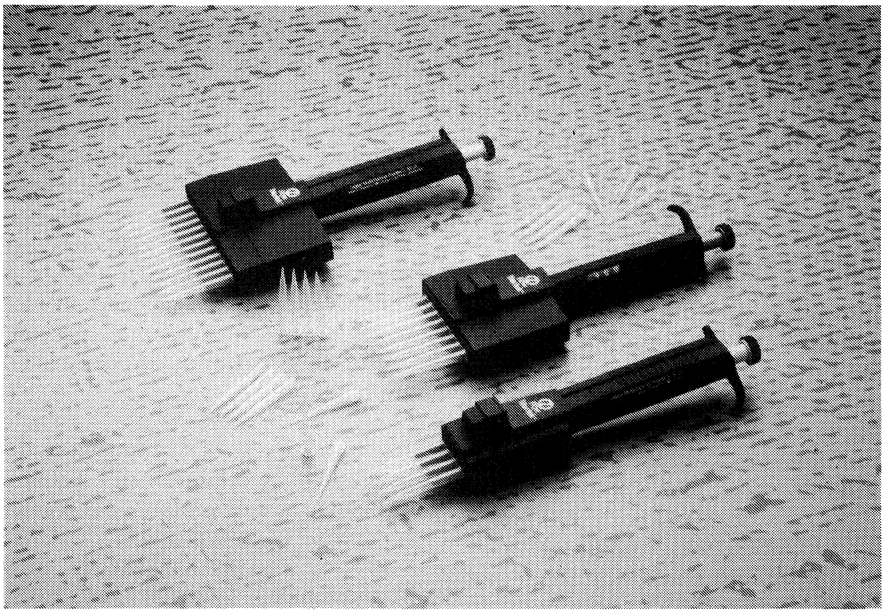

*Flow Laboratories' multichannel pipettes which offer preset volume control and digital display. The pipettes are priced from £198.*

ing service costs to a minimum and ensuring accurate dispensing every time. The pipettes are available in two colour coded volume ranges: yellow for the 5–50 μl (0.5 μl incremental steps) and green for the 50–300 μl range (5 μl incremental steps).

*More information from Woodcock Hill, Harefield Road, Rickmansworth, Hertfordshire WD3 1PQ, UK. Tel.: 0923 774666; fax: 0923 777005.*

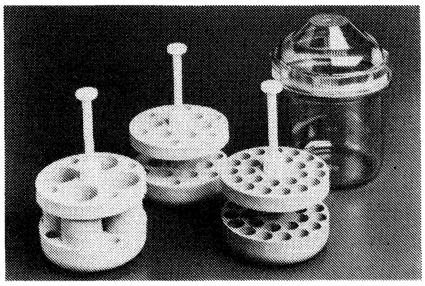

*Transparent 'Aerosolve' cannisters for Beckman TJ-6 and GP centrifuges provide increased protection by minimizing the chance of contamination from aerosols should a tube break during centrifugation. The accessory holds tubes of 3 ml capacity, and the cannister can be used as a 500 ml wide-mouth bottle. Details from Beckman at 2500 Harbor Boulevard, Fullerton, California 92634-3100, USA.*

### Array protein chemistry

Beckman's Array Protein Chemistry System has been tested in the Immunology Department of the Royal United Hospital in Bath, UK, where it has freed the staff of a laboratory where the workload continues to increase steadily for more technical duties, and has allowed samples from many different sources to be managed efficiently and at optimum speed. At Bath the Array was used largely for immunoglobulins and complement, Rh Factor, C-Reactive protein and Alpha 1 antitrypsin studies. The laboratory is a reference unit for the Royal Hospital for Rheumatic Diseases; samples are also received from two busy clinics each month, and, often in emergency situations where a 'STAT' method is demanded of the Array, the laboratory copes with regular C-Reactive protein estimations on leukaemic patients and those undergoing marrow transplant operations.

As the Royal United is a district general hospital covering a wide area and providing a service for no less than 28 outlying hospitals and about 500 local GPs, the workload is substantial for the 4.5 staff working in the only Immunology Department in the district. The laboratory found Beckman's support on Array very effective, with back-up information and service readily available. An additional advantage was considered to be the simplicity of maintenance on the Array.

*Further information about the Array from Beckman, Progress Road, Sands Industrial Estate, High Wycombe, Buckinghamshire, UK. Tel.: 0494 441181.*

### LIMS interfacing

Beckman's IDAS (Instrument Data Acquisition System) was designed for the Beckman CALS Lab Manager, but it is fully compatible with other systems and permits the interfacing of a laboratory computer to practically every laboratory instrument with RS-232 output. Complete two-day communication between instrument and host is achieved through the use of a special Laboratory Interface Language (LIL) and Digimetry MK5 instrument coupler.

The microprocessor-controlled instrument coupler provides a number of vital interactive communication functions. An operator can use the keypad and function keys on the MK5 to select instruments, select tests and enter sample identification, or, as data is collected to review, accept and process it before transmitting it to the host computer for storage and processing.

An advantage of the IDAS system over custom software packages is its flexibility: IDAS applications are readily moved between supported computer systems – so it should continue to perform effectively despite operating system changes.

*Further information from Beckman, Progress Road, Sands Industrial Estate, High Wycombe, Buckinghamshire, UK. Tel.: 0494 441181.*

### PSA's TouchStone software expanded

The PSA TouchStone software was developed to provide automatic control data capture and manipulation for the PSA Merlin Plus Systems. It is an IBM compatible software system which utilizes a U-Micro DIO card to set up the equipment included in any system.

The instruments involved typically include an autosampler, PSA vapour generator and a fluorescence detector. The menu-driven software sets up and controls these instruments and continually checks their performance. The output derived from the fluorescence detector is on a continuous flow basis and the measurements are collected as peak height or peak areas or even time sliced integration on the peak plateau.

For many techniques in analytical chemistry, such as flow-injection analysis and electrothermal vaporization, a transient signal is produced in contrast to the continuous level. The PSA TouchStone software has been extended to handle these signals and to control the PSA 40.630 12-Port Teflon rotary valve assembly. With this unit, which is plug-compatible with the PSA hydride generator, the user is able to switch from hydride generation sample introduction to flow-injection principles. This is accomplished in a simple fashion by setting up the method template. The sample is withdrawn from the autosampler into the loop of the Teflon valve, after a predefined time the sample is fed into the sample input stream, the transient signal monitored, and after a set period the valve is returned for the next sample.

The full facilities include sample Tag number, reporting facilities or extension to 'hands off' analysis using the FPSA 20.080 Autosampler.

*For further details contact P S Analytical Ltd, Arthur House, B4 Chaucer Business Park, Watery Lane, Kemsing, Sevenoaks, Kent TN15 6QY, UK. Tel.: 0732 63416; fax: 0732 61340.*

### Peptide synthesizer

The BT 7600 peptide synthesizer incorporates a device that detects and monitors coupling reaction rates by responding to conductivity changes in the sample solution. This conductivity sensor tracks and controls the coupling reaction and deprotection steps of the synthesis, providing a simple, accurate, non-destructive means of monitoring the progress of synthesis reactions and determining when they are complete. The sensor is economical, offering significant savings both in time and reagent use, and gives high coupling efficiencies. It predicts the time required for the coupling reaction, and suspends the synthesis if it is not completed within this time so that the operator can take appropriate action. It provides unrivalled accuracy on the reaction rate of any scale of synthesis from 0.05 to 1 mole of peptide.

The BT 7600 is controlled by a built-in computer under MS-DOS. Menu-driven software makes operation simple, and methodologies may be set up and stored on disc. Under computer control, all reagents are delivered to the reaction chamber under positive inert gas pressure from sealed, airtight bottles. The progress of the synthesis is shown in real time on a full-colour display.

The system allows up to eight amino-acids to be coupled automatically without operator intervention. The BT 7600 and its conductivity sensor are optimized for FMOC opfp esters, and can handle modified and unusual amino-acids. Peptides may also be synthesized using other FMOC and BOC methodologies.

*Further information from Biotech Instruments Ltd, 183 Camford Way, Luton, Bedfordshire LU3 3AN. Tel.: 0582 502388.*

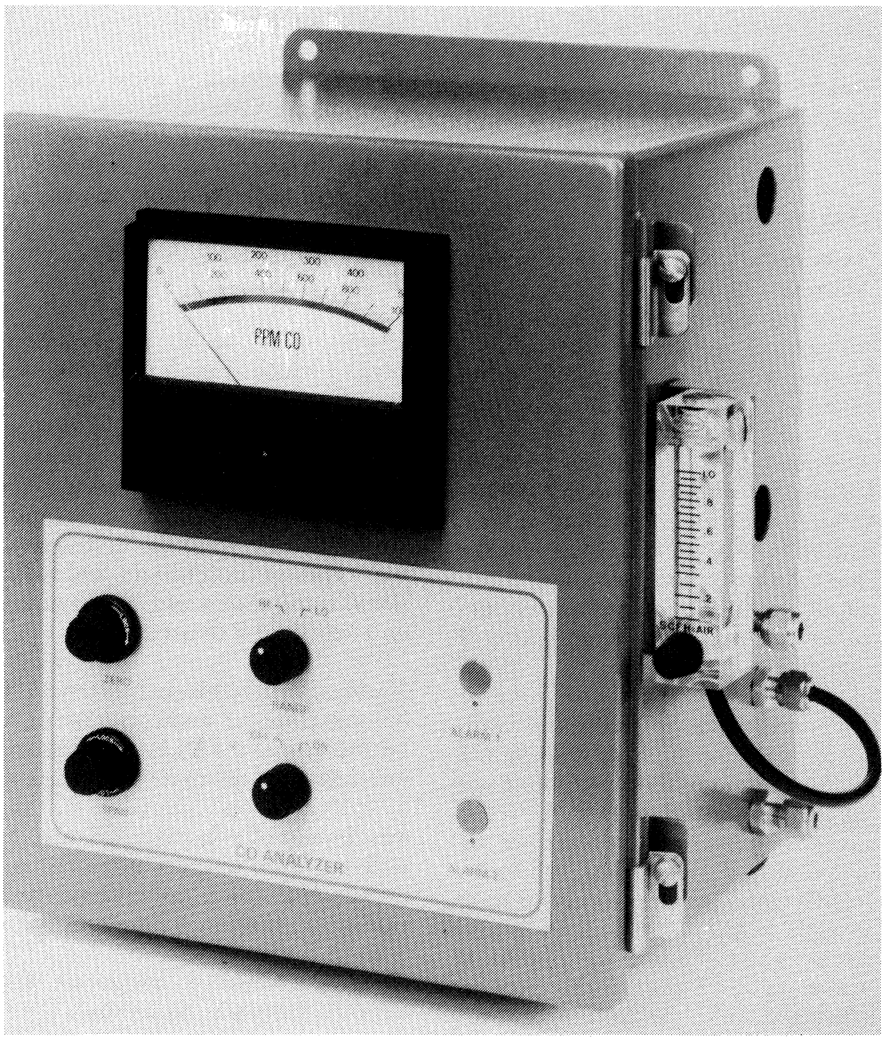

*Teledyne has recently announced a continuous on-line carbon monoxide analyser. The Model 826 features measuring ranges of 0-500/0–100 parts-per-million (ppm). Optional ranges as low as 0–100 ppm and as high as 0–2.5% are available. The analyser uses Teledyne's F-series elecrolyte and features +2% full-scale accuracy with 90% response in less than 30 seconds. A data-sheet is available from Teledyne Analytical Instruments, The Harlequin Centre, Southall Lane, Southall, Middlesex UB2 5NH, UK.*

### Electronic thermometer

Hanna have announced a new electronic thermometer called 'Ercole' which will be suitable for temperature monitoring in offices, computer rooms, factories, and hospitals and for laboratory and process applications. The light-weight Ercole has a built-in sensor for ambient temperature measurement over the range −10 to +60°C. The Ercole also has a permanently connected remote probe with a 1 metre cable which measures from −20 to +70°C and is suitable for air and liquid temperature measurement. The Ercole allows an upper and lower temperature limit to be pre-set and if the temperature is exceeded an audible alarm is triggered. A digital clock is another standard feature of the thermometer.

*The Ercole is priced at £22.00 and is available from Hanna UK, Happy Valley Industrial Park, Primrose Hill, Kings Langley, Hertfordshire WD4 8HZ, UK. Tel.: 09277 60655.*

### Oxygen alarm

Teledyne's Model 335 Oxygen Monitor quickly and accurately measures the oxygen concentration in control rooms, closed atmospheres, critical breathing circuits and other situations that require the fail-safe continuous monitoring of ambient air. This AC-powered instrument is provided with a power-failure back-up consisting of two NiCad battery packs maintained on a 'trickle' charge. Primary power interruption and subsequent battery operation is signalled by illumination of the LO AC panel light.

Two oxygen alarm levels are available: *caution* (19% 02) and *danger* (17 1/2% 02). Both alarm setpoints are customer specified and factory set to prevent tampering or resetting by unauthorized personnel. When an insufficient oxygen concentration triggers either alarm, both a red panel light and audible annunciator are energized. The alarms remain energized until the oxygen concentration has been elevated above the trigger point.

*Details from Teledyne Analytical Instruments, The Harlequin Centre, Southall Lane, Southall, Middlesex UB2 5NH, UK. Tel.: 01 571 9596; fax: 01 571 9439.*

### Titrator

Up to four burettes can be mounted on Mettler's new DL70 Titrator. Automation can thus be extended with several burettes without the need for more space. As a multitasking system, the titrator controls burettes and electrodes automatically. Parallel to this, new sample data can be entered, a new method developed, or modifications made to a method currently in progress.

Each method is composed of a series of functions, such as sample preparation, content determination, evaluation and result recording. The functions can be freely defined by the user, allowing a practically unlimited field of applications.

The DL70 handles most common titration methods, such as acid/base, redox, complexation and precipitation titrations to equivalence or end-point. Special applications such as pH-stating or titrations according to standards (DIN, ASTM) can also be performed.

Operation is via an alphanumeric keypad which is used to identify the sample, configure methods and enter instructions. The multiline display with graphics capability provides continuous information during the titration, including online titration curves and curve of the first derivative. These titration data and additional information can be displayed on a terminal if desired.

Various peripherals such as the ST20 Sample Changer and computers can be attached to the DL70. An RS232C interface for a dot matrix printer and a current loop interface for the attachment of a Mettler balance, as well as three outputs for auxiliary instruments such as pumps and valves, are built in a standard.

*Details from Mettler Instrumente AG, CH-8606 Greifensee, Switzerland.*

### Reverse osmosis system

An American automobile manufacturer has discovered a cost-effective solution to high deionized (DI) water costs in a chromate rinse for automobile parts – the OSMO®-43B reverse osmosis (RO) system by Osmonics.

Automobile parts are dipped in treatment solutions of phosphate and then chromate to clean and prepare the metal surfaces prior to an electrodeposition paint bath. After the chromate treatment, the parts are submerged in two rinse baths, followed by two DI spray rinses. The DI water used in this spray rinse is often sent to drain.

However, at this location an OSMO-43B machine takes the water from the first DI spray rinse, lowers its conductivity level, and returns the DI water to a second spray rinse. A key benefit to the system is that the volume of wastewater sent to drain from the second spray rinse is greatly reduced by as much as 90%.

A typical chromate spray rinse uses nearly 100 000 gallons of DI water per day at a cost of roughly $500 per day ($5.00/1000 gallons) or $125 000 per year (250 working days). The OSMO-43B RO system can save over $100 000 per year by reclaiming valuable DI rinse water sent to drain.

*Osmonics can help manufacturers using ED paint processes to face the challenge of high DI water costs. For further information on OSMO reverse osmosis systems contact OSMO Engineered Products and Systems, Osmonics, Inc., 5951 Clearwater Drive, Minnetonka, Minnesota 55343, USA. Tel.: 612 933 2277; fax: 612 933 0141*

### Electro–hydraulic mounting press

Buehler have introduced a mounting press for sample preparation applications. The Simplimet 3 is an automatic electro-hydraulic mounting press that brings fast production of quality mounted samples to the microstructural analysis laboratory. All Simplimet 3 presses incorporate a number of state-of-the-art features, including corrosion and impact resistant reaction-injection-moulded cabinetry, a reliable electro-hydraulic system for applying and maintaining selected pressure, electronic touch-pad switches for selection and automatic control of all moulding cycle parameters, including temperature, pressure and time, and digital LED parameter read-outs and bar graphs. In addition, the Simplimet 3 mounting press accommodates a range of mould sizes from 25mm to 1-1/2in and incorporates a water-cooled mould-cooling unit to produce cool mounts in seven to nine minutes.

*Further information from Buehler UK Ltd, PO Box 150, Binns Close, Coventry CV4 9XJ, UK. Tel.: 0203 471411.*

### Solvector

As a further step in increasing Cavendish's expertise and range of products available in the field of solvent vapour detection, a new version of the Solvector has recently been launched. This can operate as a continuous-reading instrument or with the GC attachment to give a specific response to one solvent. This is an advance over the other photoionization detection systems available and is priced competively.

*Details from Cavendish Applied Technology Ltd, Unit 24, Clifton Road, Cambridge CB1 4ZD, UK.*

### PC-based chromatography workstation

Compatible with gas or liquid chromatographs from any manufacturer, the new HP 3365 ChemStation is a personal computer functioning as a fully featured chromatography workstation. The MS-DOS based analytical software, which includes data reduction algorithms for precise quantitative results, will run on the HP Vectra PC or any other PC/AT compatible. Used with the HP Vectra, it is the only single-vendor system of its kind.

The software uses Microsoft® Windows Presentation Manager to provide a mouse-driven, graphical environment with multitasking capabilities and multiple windows. Pull-down menus, dialogue boxes and icons simplify operation, and as the software runs under MS-DOS, results may be ported to word-processors, spreadsheets and other standard office packages.

Many such tasks can be performed simultaneously through the use of MS-Windows, which also allow several chromatograms to be displayed and compared on screen. Windows may be enlarged, reduced, overlapped or moved.

The HP 3365 ChemStation may be connected directly to the HP 5890 Series II GC for full instrument control and data acquisition. It is fully multitasking, and can operate

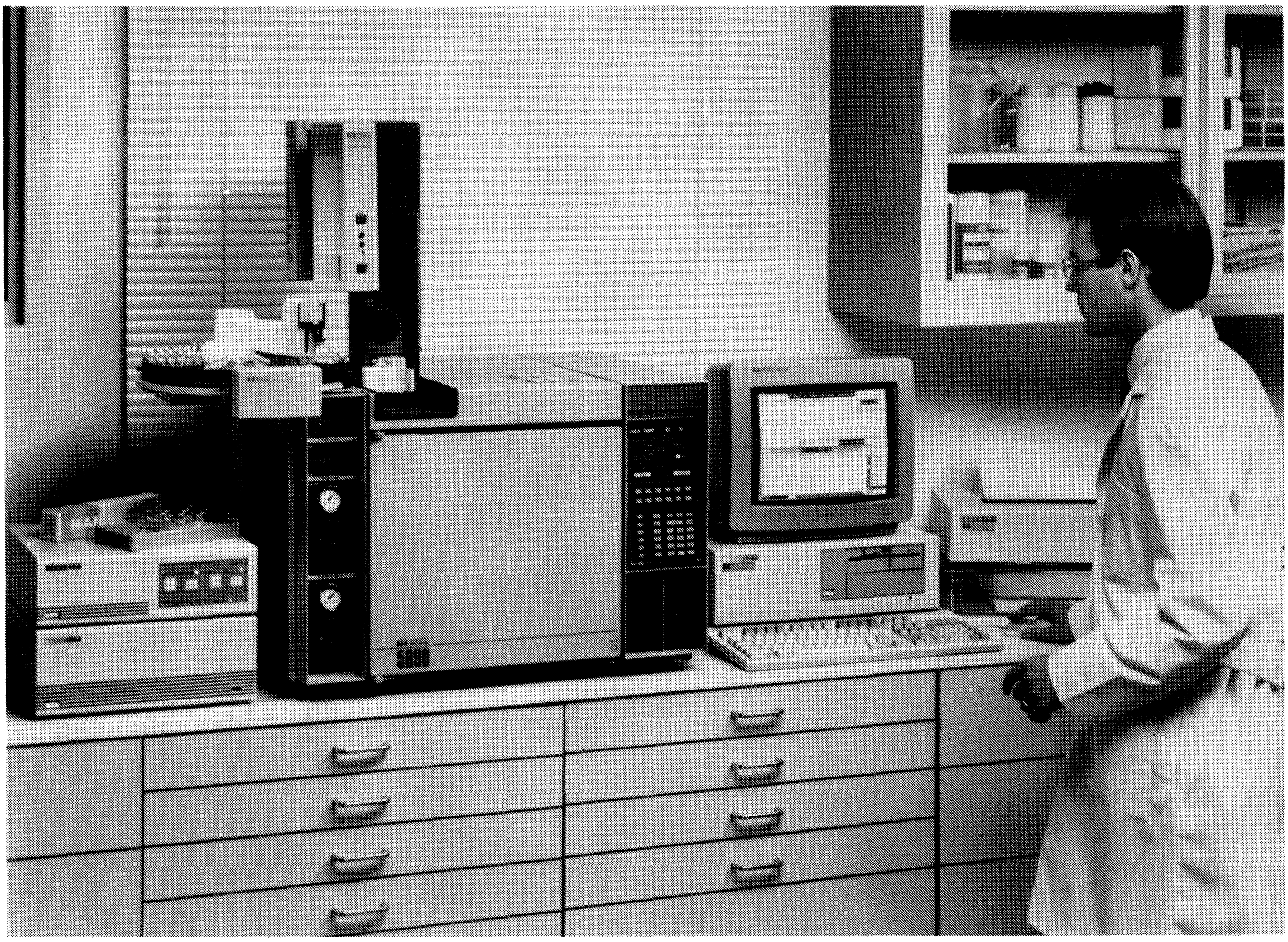

*The HP 3365 chromatographic ChemStation from Hewlett-Packard. The workstation runs on the HP Vectra PC and can control single or multiple chromatographic instruments. With it is the HP 5890 Series II gas chromatograph.*

several GCs simultaneously. It may also be connected, via the HP 35900C dual-channel interface, to any other manufacturer's liquid or gas chromatograph for single or multiple instrument data acquisition.

As a PC-based system, the HP 3365 is compatible with all standard interfaces. It may be linked to other PCs and can share such peripherals as printers and data storage devices, saving both space and cost.

The ChemStation can be networked to laboratory data systems. It is fully compatible with HP 1000 and HP 3000 minicomputers, as well as IBM and DEC systems. It may also be linked into HP LABSAM and other commercially available LIMS systems.

*Enquiries to Analytical Products Group, Hewlett-Packard Ltd, King Street Lane, Winnersh, Wokingham, Berkshire RG11 5AR, UK. Tel.: 0734 784774.*

